# Role of the gut microbiota in cefoperazone/sulbactam-induced epilepsy in mice with chronic renal failure

**DOI:** 10.1080/0886022X.2024.2371551

**Published:** 2024-06-28

**Authors:** Yulu Wu, Donghua Gu, Jie Li, Jing Li, Guocun Hou

**Affiliations:** a Department of Nephrology, Suzhou Hospital, Affiliated Hospital of Medical School, Nanjing University, Suzhou, China; b Department of Pathology, Suzhou Hospital, Affiliated Hospital of Medical School, Nanjing University, Suzhou, China; c Department of Neurology, Suzhou Hospital, Affiliated Hospital of Medical School, Nanjing University, Suzhou, China

**Keywords:** Cefoperazone/sulbactam, gut microbiota, chronic kidney disease, epilepsy

## Abstract

**Objectives:**

The mechanism of cefoperazone/sulbactam-induced epilepsy in chronic kidney disease (CKD) patients is not yet clear. We hypothesized that cefoperazone/sulbactam-induced epilepsy could be based on two main factors: neurotoxicity caused by drug accumulation after renal failure and an abnormal gut microbiota (GM).

**Methods:**

A chronic renal failure (CRF) model in mice was established, and then different doses of cefoperazone/sulbactam were injected to induce epilepsy in mice. Normal mouse feces for fecal microbiota transplantation (FMT) were collected. We observed the changes in feces, mental state, and activity of each group of mice. After killing, we collected kidneys and colon for H&E staining. We collected mouse feces for the 16S RNA sequencing of bacteria.

**Results:**

All CRF mice injected with different concentrations of cefoperazone/sulbactam experienced grade-V seizures and eventually died, whereas normal control mice did not. However, after FMT intervention, the time of epilepsy onset and death in mice was delayed. Early FMT intervention resulted in more mice surviving (*p* = .0359). Moreover, the villi in the mucosal of group-CS layer fell off, goblet cells missed, and crypts disappeared. The mucosal layer and submucosa were clearly separated. The morphology of intestinal tissue of the CFS and FS group was improved. After FMT, the changes of the GM were observed.

**Conclusions:**

The GM may be involved in the epilepsy induced by cefoperazone/sulbactam in CRF mice. FMT can delay the onset of epilepsy in CRF mice induced by cefoperazone/sulbactam, and the earlier the intervention, the better the effect.

## 1. Introduction

Cefoperazone/sulbactam is a combination drug used as an antibiotic. It is metabolized mainly by the liver and kidneys. The permeability of this combination drug through the blood–brain barrier is poor. Cefoperazone/sulbactam can be cleared by hemodialysis, but a risk of accumulation in the body during intervals between dialysis treatments remains. A high concentration of this drug combination in the blood can penetrate the blood–brain barrier, thereby affecting the metabolism of brain cells and triggering a series of neurologic and psychiatric symptoms.

We have observed that some patients with stage-5 chronic kidney disease (CKD) may experience limb twitching, closed jaws, dilated eyes, and unclear consciousness 7–10 days after use of cefoperazone/sulbactam, suggesting an epileptic seizure. Research [1] has suggested that cefoperazone/sulbactam causes neurologic symptoms (e.g., epilepsy) in patients with renal insufficiency by four main mechanisms. First, patients with renal insufficiency often have complications such as anemia and hypoproteinemia. These complications result in a decrease in the rate of binding of the drug and proteins in plasma, and an increase in free-drug concentration, causing drug accumulation in the body and central nervous system (CNS) symptoms. Second, if renal dysfunction occurs, the glomerular filtration rate decreases, and the half-life of sulbactam is prolonged. Third, the accumulation of cefoperazone/sulbactam can cause the deposition of drug metabolites in brain tissue through the blood–brain barrier. The fourth mechanism is an imbalance in the gut microbiota (GM) [2].

We hypothesized that cefoperazone/sulbactam-induced epilepsy could be based on two main factors: neurotoxicity caused by drug accumulation after renal failure and an abnormal GM. We explored: (i) the nature of cefoperazone/sulbactam-induced epilepsy in CRF mice; (ii) levels of biomarkers that may be reduced when using antibiotics in patients suffering from uremia; (iii) whether fecal microbiota transplantation (FMT) could reduce the onset of epilepsy.

## 2. Methods

### 2.1. Ethical approval of the study protocol

The study protocol was approved (2023-A22) by the Experimental Animal Ethics Committee of Suzhou Institute of Biomedical Engineering Technology (Suzhou, China) within the Chinese Academy of Sciences.

### 2.2. Establishment of a CRF model in mice

C57BL6 mice (6–8 weeks) were divided randomly into two groups. They were fed adaptively for 1 week and their bodyweight recorded. The normal (control) group (N, *n* = 11) continued to receive normal feed. The CRF (model) group (C, *n* = 20) was fed chow containing 0.2% adenine [3]. Mice were weighed weekly, and we observed changes in urine volume and stool characteristics. Mice were killed at week 4. Blood, urine, and kidneys were collected immediately. Levels of blood urea nitrogen (BUN) and serum levels of creatinine (Scr) were recorded. We weighed the kidneys, calculated the renal index (kidney weight/bodyweight × 100%), and undertook hematoxylin and eosin (H&E) staining of the kidney.

### 2.3. Induction of epilepsy in mice with CRF

A total of 12 mice were used as pre-experimental mice to explore the dosage of cefoperazone sulbactam sodium. After 4 weeks, mice suffering from CRF (hereafter termed ‘CRF mice’) were divided randomly into four groups (control (C), low-dose (L), medium dose (M), and high-dose (H)) (*n* = 3), and were injected (i.p.) with physiologic (0.9%) saline, cefoperazone/sulbactam (400 mg/kg/d), cefoperazone/sulbactam (1000 mg/kg/d), and cefoperazone/sulbactam (2000 mg/kg/d), respectively, at a fixed time for 10 days. The injection dose [4] of cefoperazone/sulbactam is a combination dose. The stool characteristics, prevalence of epilepsy, and survival of mice were documented. The kidneys and colon of mice were collected for H&E staining.

### 2.4. FMT

After 4 weeks of feeding, feces were obtained from the normal group of mice: one hand wore sterile gloves, the other hand grabbed the tail of the mice, disinfected the mice’s anus with a 75% alcohol cotton ball, gently massaged the mice’s abdomen to stimulate defecation, and used sterile forceps to take the new feces of the mice in a 15 mL sterile centrifuge tube.

Mouse feces (200 mg) were placed in a sterile container. Then, 5 mL of 0.9% saline was added for dissolution. The feces solution was mixed evenly, centrifuged (888 × *g*, 10 min, 4 °C) to obtain the supernatant, followed by freezing at −80 °C. After thawing at room temperature, 200 µL was administered to each mouse by gavage at a fixed time. One group of mice (F, *n* = 15) underwent FMT immediately after receiving adenine-containing feed and, after 4 weeks, 0.9% saline was injected (i.p.) for 10 days. We observed changes in the feces, mental state, and activity of mice.

### 2.5. 16S RNA sequencing of bacteria

*Sample collection*: Seven mouse fecal samples were collected, including seven groups (N, NS, C, CS, CSF, F, and FS). Packaged and sent them to Sangon Biotech (Shanghai) Co., Ltd. (Shanghai, China) for 16S rDNA sequencing.

*Deoxyribonucleic acid (DNA) extraction*: Total community genomic DNA extraction was performed using a E.Z.N.A™ Mag-Bind Soil DNA Kit (Omega, M5635-02, Norcross, GA), following the manufacturer’s instructions. We measured the concentration of the DNA using a Qubit 4.0 (Thermo, Waltham, MA) to ensure that adequate amounts of high-quality genomic DNA had been extracted.

*16S ribonucleic acid (RNA) gene amplification by polymerase chain reaction (PCR)*: Our target was the V3–V4 hypervariable region of the bacterial 16S rRNA gene. PCR was started immediately after the DNA was extracted. The 16S rRNA V3–V4 amplicon was amplified using 2 × Hieff^®^ Robust PCR Master Mix (Yeasen, 10105ES03, Shanghai, China). Two universal bacterial 16S rRNA gene amplicon PCR primers (PAGE purified) were used: the amplicon PCR forward primer (CCTACGGGNGGCWGCAG) and amplicon PCR reverse primer (GACTACHVGGGTATCTAATCC). The reaction was set up as follows: microbial DNA (10 ng/µL) 2 µL; amplicon PCR forward primer (10 µM) 1 µL; amplicon PCR reverse primer (10 µM) 1 µL; 2 × Hieff^®^ Robust PCR Master Mix (Yeasen, 10105ES03, Shanghai, China) (total 30 µL). The plate was sealed and PCR was performed in a thermal instrument (Applied Biosystems 9700, Foster City, CA) using the following program: one cycle of denaturing at 95 °C for 3 min, first five cycles of denaturing at 95 °C for 30 s, annealing at 45 °C for 30 s, elongation at 72 °C for 30 s, then 20 cycles of denaturing at 95 °C for 30 s, annealing at 55 °C for 30 s, elongation at 72 °C for 30 s, and a final extension at 72 °C for 5 min. The PCR products were checked using electrophoresis in 2% (w/v) agarose gels in TBE buffer (Tris, boric acid, and EDTA) stained with ethidium bromide (EB) and visualized under UV light.

*16S gene library construction, quantification, and sequencing*: We used Hieff NGS™ DNA Selection Beads (Yeasen, 10105ES03, Shanghai, China) to purify the free primers and primer dimer species in the amplicon product. Samples were delivered to Sangon BioTech (Shanghai) (Shanghai, China) for library construction using universal Illumina adaptor and index. Before sequencing, the DNA concentration of each PCR product was determined using a Qubit^®^ 4.0 Green double-stranded DNA assay and it was quality controlled using a bioanalyzer (Agilent 2100, Santa Clara, CA). Depending on coverage needs, all libraries can be pooled for one run. The amplicons from each reaction mixture were pooled in equimolar ratios based on their concentration. Sequencing was performed using the Illumina MiSeq system (Illumina MiSeq, San Diego, CA), according to the manufacturer’s instructions.

Seven mouse fecal samples were collected, including seven groups (N, NS, C, CS, CSF, F, and FS). Packaged and sent them to Sangon Biotech (Shanghai) Co., Ltd. (Shanghai, China) for 16S rDNA sequencing. After extracting the genomic DNA of fecal bacteria, the raw data are obtained through PCR amplification, purification of amplification products, and machine sequencing. The sequencing data are analyzed and compared using the Bioinformatics Cloud i-Sanger platform, including statistical analysis of species abundance of each sample at different classification levels, analysis of the community composition of GM, and species differences between GM in each group of samples.

### 2.6. Effect of FMT on cefoperazone/sulbactam-induced epilepsy

After 4 weeks of feeding, mice in groups N, C, and F were injected (i.p.) with cefoperazone/sulbactam (1000 mg/kg/d) for 10 days, and labeled as ‘NS’, ‘CS’, and ‘FS’ groups (*n* = 5), respectively. After feeding group-C mice for 4 weeks, cefoperazone/sulbactam (1000 mg/kg/d) was injected (i.p.), and a fecal suspension was administered by gavage daily for 10 days; this was labeled the ‘CSF’ group. We observed the changes in feces, mental state, and activity of each group of mice. After killing, we collected kidneys and colon for H&E staining. We collected feces for the 16S RNA sequencing of bacteria.

### 2.7. Statistical analyses

Data were analyzed using Prism (GraphPad, La Jolla, CA) and subjected to tests on a normal distribution and homogeneity of variance. Quantitative data are expressed as the mean ± standard deviation. Comparisons of data between multiple groups were conducted using one-way analysis of variance (ANOVA). Comparisons of data between multiple groups at the same time point were conducted using two-way ANOVA. Survival analysis was conducted using the log-rank test. *p* < .05 was considered significant.

To assess sample adequacy, rarefaction curves of the observed numbers of operational taxonomic unit (OTU) were constructed. The OTU rarefaction curve and rank abundance curves were plotted in R(version 3.6.0) (R Foundation for Statistical Computing, Vienna, Austria). Beta diversity evaluates differences in the microbiome among samples and is normally combined with dimensional reduction methods such as principal coordinate analysis (PCoA), non-metric multidimensional scaling (NMDS), or constrained principal component analysis (PCA) to obtain visual representations. These analyses were visualized using R vegan package (version 2.5-6), and finally the inter-sample distances were presented as scatterplots.

## 3. Results

### 3.1. Features of mice

#### 3.1.1. Bodyweight

The bodyweight (in g) of group-N mice maintained an increasing trend, whereas the bodyweight of group-C and group-F mice continued to decrease. Compared with group N, the bodyweight loss in group C and group F was significant (*p* < .001); at weeks 1, 2, and 4, there was no significant difference between group C and group F (*p* = .643 at week 1, *p* = .16 at week 2, *p* = .099 at week 4); at week 3, there was a significant difference between group C and group F (*p* = .019). These data indicated that adenine feeding could lead to bodyweight loss in mice, whereas FMT intervention did not have a significant effect on the bodyweight of CRF mice.

#### 3.1.2. Food intake

The food intake (in g/day) of group-N mice increased first and then decreased slowly, whereas the food intake of group-C and -F mice decreased significantly. From week 1 to week 4, compared with group N, there was a significant decrease in food intake in groups C and F (*p* < .001). There was no significant difference between group C and group F (*p* = .809 at week 1, *p* = .292 at week 2, *p* = .22 at week 3, and *p* = .382 at week 4). These data indicated that adenine feeding could lead to a decrease in food intake in mice, whereas FMT intervention had no effect on food intake in CRF mice.

#### 3.1.3. Water intake

There was no significant change in the water intake (in mL/day) of group-N mice, whereas the water consumption of group-C and -F mice increased. Compared with group N, there was a significant increase in water intake in group C (*p* = .045 at week 1, *p* = .004 at week 2, and *p* < .001 at weeks 3–4). Compared with group N, there was a significant increase in water intake in group F (*p* = .049 at week 1, *p* = .003 at week 2, and *p* < .001 at weeks 3–4). There was no significant difference between group C and group F (*p* = .786 at week 1, *p* = .52 at week 2, *p* = .123 at week 3, and *p* = .166 at week 4). These data suggested that adenine feeding could lead to excessive water intake in mice, whereas FMT intervention had no effect on the water intake of CRF mice.

#### 3.1.4. Physiological activity

The mental activity of group-N mice was normal, and they had shiny black fur and no depilation. The color and humidity of their feces were normal. Some mice in group C and group F experienced mental fatigue, reduced activity, and hair loss. The stools of group-C mice were gray and dry. The color and humidity of the stools of group-F mice were normal. These data suggested that adenine feeding could affect the physiological activity of mice and lead to changes in their stool characteristics.

#### 3.1.5. Survival

From establishment of the model to the end of the experimental observation (8 weeks), all mice in N, C, and F groups survived. These results suggested that establishment of a mouse model of CRF using feed laced with 0.2% adenine did not affect the survival of mice, and that FMT did not affect the survival of CRF mice.

#### 3.1.6. Serum biochemical indicators

Compared with group-N (*n* = 6) mice, the BUN level and serum creatinine level in group-C (*n* = 5) and group-F (*n* = 5) mice were increased significantly (*p* < .001). There was no significant difference between group C and group F in the BUN level (*p* = .802) and serum creatinine level (*p* = .597). These data indicated that a mouse model of chronic renal failure (CRF) had been established, and that FMT had no effect on establishment of this model.

#### 3.1.7. Changes in anatomic appearance of the kidney and renal index

Mice were killed at week 4. The kidneys of group-N (*n* = 6) mice were dark-red with a smooth surface, whereas those of group-C (*n* = 5) and group-F (*n* = 5) mice were gray-white with surface particles and reduced in size. The renal index of group-N mice was 1.18 ± 0.05%, whereas the renal index of group-C mice (1.32 ± 0.07%, *p* = .018) and group-F mice (1.33 ± 0.10%, *p* = .012) increased significantly. There was no significant difference between group C and group F (*p* = .852). These data suggested that adenine-laced food caused damage to the kidneys of mice, and that FMT did not alter the damage to the kidneys of CRF mice.

#### 3.1.8. Renal pathology

In group-N mice, the distribution of glomeruli was uniform (with no atrophy or proliferative changes), no dilation of renal tubules, and no infiltration of inflammatory cells in the renal interstitium. In group C, the renal tubules were dilated significantly, with infiltration of interstitial inflammatory cells, degeneration (turbid and swollen) of renal tubular epithelial cells, necrosis of some renal tubular epithelial cells, purulent tubular cells, and mild hyperplasia of renal cortical interstitial fibrous tissue. Essentially, group F was the same as group C in terms of renal pathology.

#### 3.1.9. Colon pathology

The morphology of intestinal tissue of group-N, -C, and -F mice was normal. This was manifested as long and intact villi and an orderly arrangement of intestinal cells without significant changes.

### 3.2. Cefoperazone/sulbactam-induced epilepsy in CRF mice

According to the Racine Seizure Scale, the seizure grading of mice (in levels) was: 0 (normal behavior); I (gazing, facial twitching, head swinging, chewing); II (rhythmic nodding accompanied by angular chewing); III (forelimb clonus); IV (bilateral forelimb clonus with hind limbs standing like ‘kangaroos’); V (general convulsion, loss of balance, and falling).

CRF mice were administered a low dose (400 mg/kg/d, group L), medium dose (1000 mg/kg/d, group M), or high dose (2000 mg/kg/d, group H) of cefoperazone/sulbactam. Mice in all groups showed varying degrees of convulsions, tail elevation, unstable walking, and mental fatigue, lasting for >10 min before finally dying. Group-L mice experienced grade-V seizures as early as five days after administration, and died 6, 10, and 14 days after administration. Group-M mice experienced grade-V seizures as early as three days after administration, and died 4, 9, and 10 days after administration. Group-H mice experienced grade-V seizures as early as three days after administration, and died 4, 4, and 8 days after administration. Normal-feeding group mice were injected with a medium dose of cefoperazone/sulbactam (group NS) and CRF mice were injected (i.p.) with physiologic saline (group C), but there were no seizures or deaths (*p* = .0127). These data suggested that cefoperazone/sulbactam could induce epilepsy and cause death in CRF mice, and that the higher the concentration, the earlier was the time of epilepsy and death. Cefoperazone/sulbactam administration did not induce epilepsy or cause death in normal-group mice.

#### 3.2.1. Effect of FMT on epilepsy induced by cefoperazone/sulbactam in mice

After establishing a mouse model of CRF, cefoperazone/sulbactam (1000 mg/kg/d) was administered (i.p.) for 10 days starting from week 4. Simultaneously, CSF and FS groups were administered (p.o.) a fecal suspension for 20 days. CS-group mice experienced grade-V seizures as early as four days after administration, and died 6, 8, 8, 9, and 9 days after administration. CSF-group mice suffered grade-V seizures as early as six days after administration, and died 7, 9, 10, 11, and 20 days after administration. FS-group mice experienced grade-V seizures as early as five days after administration, and died 9, 13, 13, and 18 days after administration, with one mouse surviving (*p* = .0359). These data suggested that FMT could delay the occurrence and death of epilepsy in CRF mice, and that the earlier the intervention, the better was the prognosis.

#### 3.2.2. Effect of FMT on renal pathology in CRF mice

The CS group showed obvious chronic inflammation, with lymphocyte infiltration, obvious proliferation of interstitial fibrous tissue, reduced renal tubular dilation, necrosis of renal tubular epithelial cells, and a pus cell type. From a morphologic perspective, the CSF group was similar to the CS group. Compared with CS and CSF groups, the FS group had a lower degree of inflammation, less necrosis of renal tubular epithelial cells, and fewer pyocytic tubular types.

#### 3.2.3. Effect of FMT on colonic pathology in mice

In the CS group, the villi in the mucosal layer fell off, a large area of goblet cells was missing, and crypts disappeared. The mucosal layer and submucosa were clearly separated. In the CSF group, some villi in the mucosal layer fell off, and the mucosal layer separated from the submucosal layer. The morphology of intestinal tissue of the FS group was, in general, normal.

#### 3.2.4. 16S RNA sequencing of fecal bacteria

The number of OTUs in normal mice ranges from 200 to 350, and the larger the value, the higher is the diversity. The number of OTUs in group-N mice was 295, whereas that in adenine-fed mice (group C = 352; group F = 336) increased. The decrease in OTU rank curves in groups C and F was smoother than that in group N, indicating that adenine-fed mice could increase their GM diversity. The OTU of mice injected with cefoperazone/sulbactam (NS group = 186, CS group = 218, FS group = 294) decreased compared with that in C and F groups, and the OTU rank curve decreased rapidly and sharply. These results indicated that injection of cefoperazone/sulbactam could increase the proportion of dominant bacterial groups in the GM of mice and reduce the diversity. FMT intervention increased the OTU in mice of the CSF group (248) and FS group (294) compared with that in the CS group, and the decrease in the OTU rank curve eased slightly, indicating that FMT could improve the changes caused by injection of cefoperazone/sulbactam.

Principal component analysis showed that the FS and CSF groups had a similar GM, but the CS group had larger spacing, indicating that there were significant differences in the GM of mice under experimental conditions. In CRF mice injected with cefoperazone/sulbactam, the proportion of *Anaeroplasma*, bacteria of the genera *Clostridium* and *Lactobacillus*, and *Limosilactobacillus* increased, whereas the proportion of bacteria from the genera *Duncaniella*, *Enterococcus*, *Staphylococcus*, and *Bosea* decreased. After FMT, the trend of changes in the abundance of the bacteria stated above reversed, indicating that these bacteria may be involved in epilepsy-induced death in mice. In addition, early intervention with FMT had a more significant effect than later intervention.

## 4. Discussion

Cefoperazone/sulbactam for injection is used widely as a composite preparation for the treatment of infections of systems (respiratory, reproductive, urinary tract), bones, joints, peritoneum, sepsis, meningitis, and other infections caused by sensitive bacteria. Common adverse events include reactions (allergic, gastrointestinal, hematological), liver damage, and kidney damage, but neurologic adverse events are relatively rare [5].

In 1970, a case of epilepsy caused by cephalosporins was first reported clinically. In 2003, Chow et al. found third-generation and fourth-generation cephalosporins could cause neurotoxicity, and that patients with CKD were more likely to be affected [6]. Most reports have focused on the neurologic reactions caused by ceftazidime and cefepime [7–9], whereas reports on the neurologic reactions caused by cefoperazone/sulbactam are scarce [1].

Our hospital department documented four cases of epilepsy during use of cefoperazone/sulbactam from the end of 2022 to the beginning of 2023. All patients were patients suffering from CKD on stage-5 hemodialysis in our hospital. After the onset of epilepsy, these patients underwent computed tomography of the cranium as well as tests for electrolytes, blood gases, renal function, blood pressure, and other tests excluding seizures caused by acute cerebrovascular accidents, electrolyte disorders, uremic encephalopathy, and hypertensive encephalopathy. After treatment such as hemodialysis combined with hemoperfusion, further seizures were not observed. Seizures were considered to be caused by the accumulation of cefoperazone/sulbactam.

**Figure 1.**
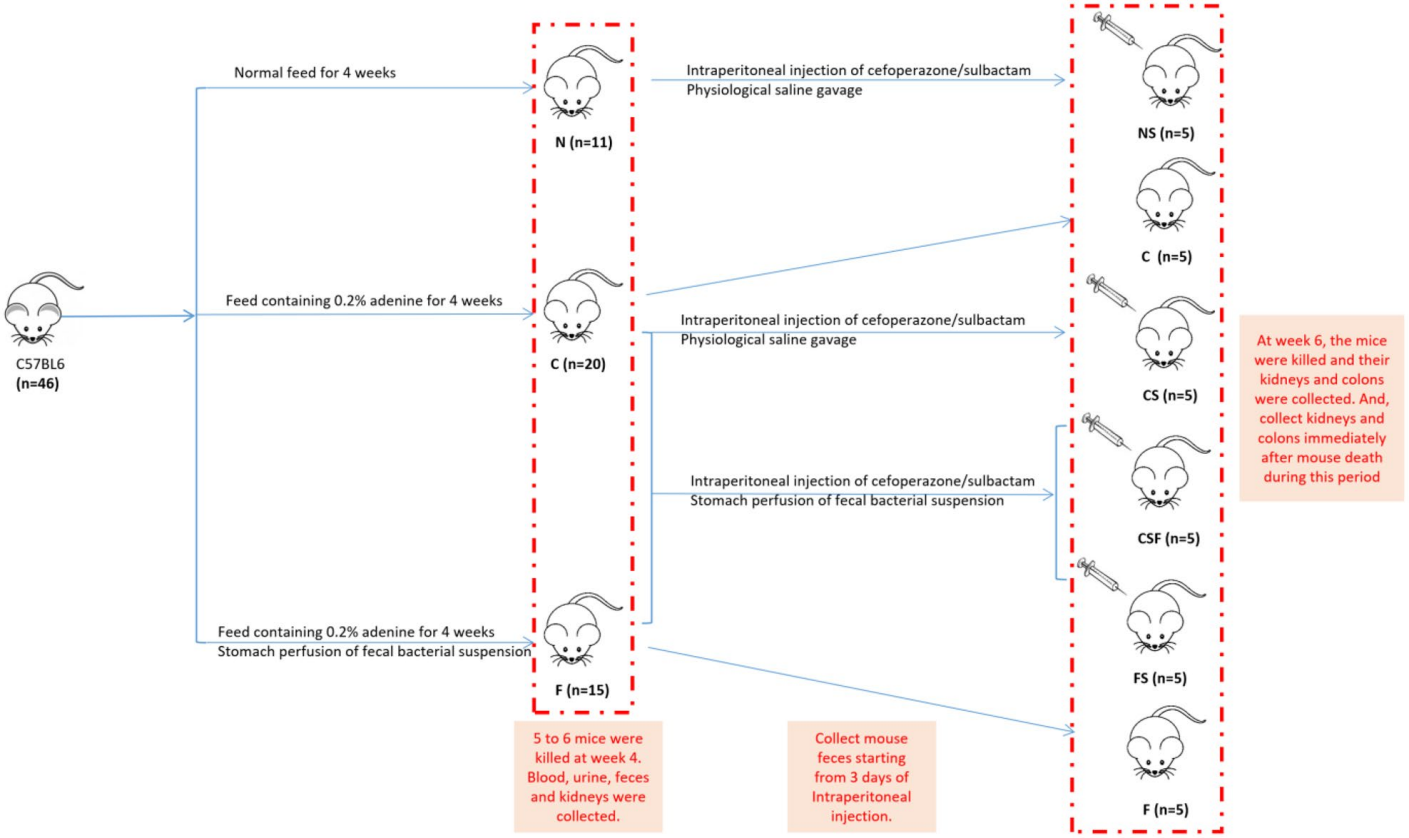
Experimental grouping.

In our research, a CRF model in mice was established, and then different doses of cefoperazone/sulbactam were injected to induce epilepsy in mice. Normal mouse feces for FMT were collected. We observed the changes in feces, mental state, and activity of each group of mice. After killing, we collected kidneys and colons for H&E staining (Figure 1).

**Figure 2.**
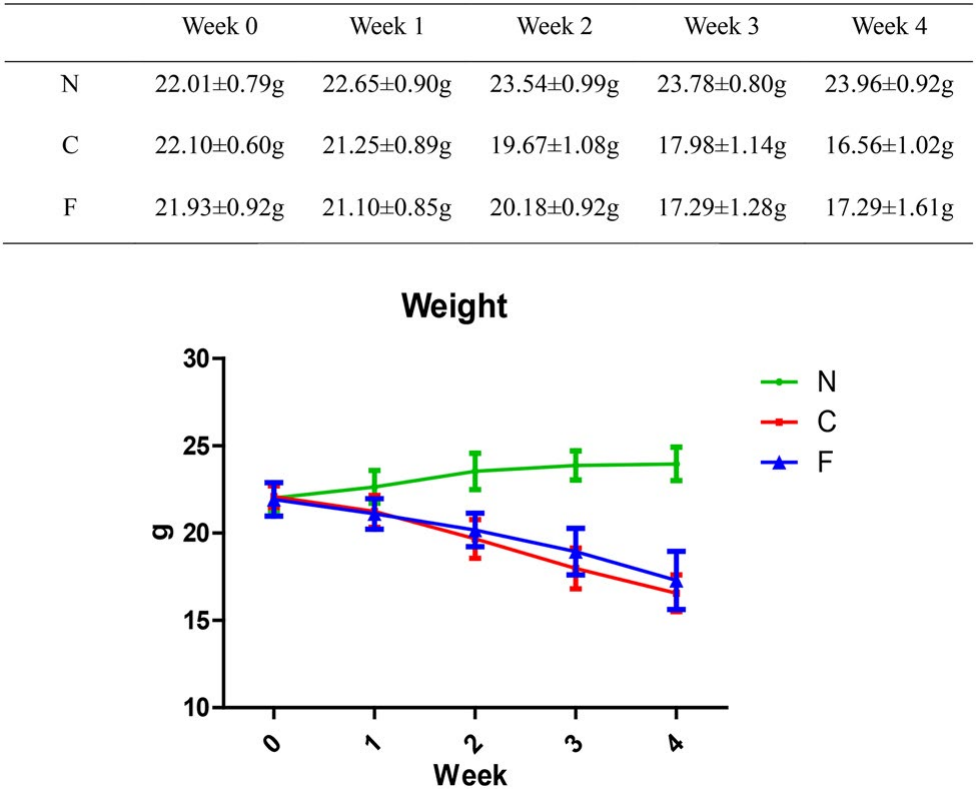
Effect of adenine and fecal bacteria transplantation on mouse bodyweight.

**Figure 3.**
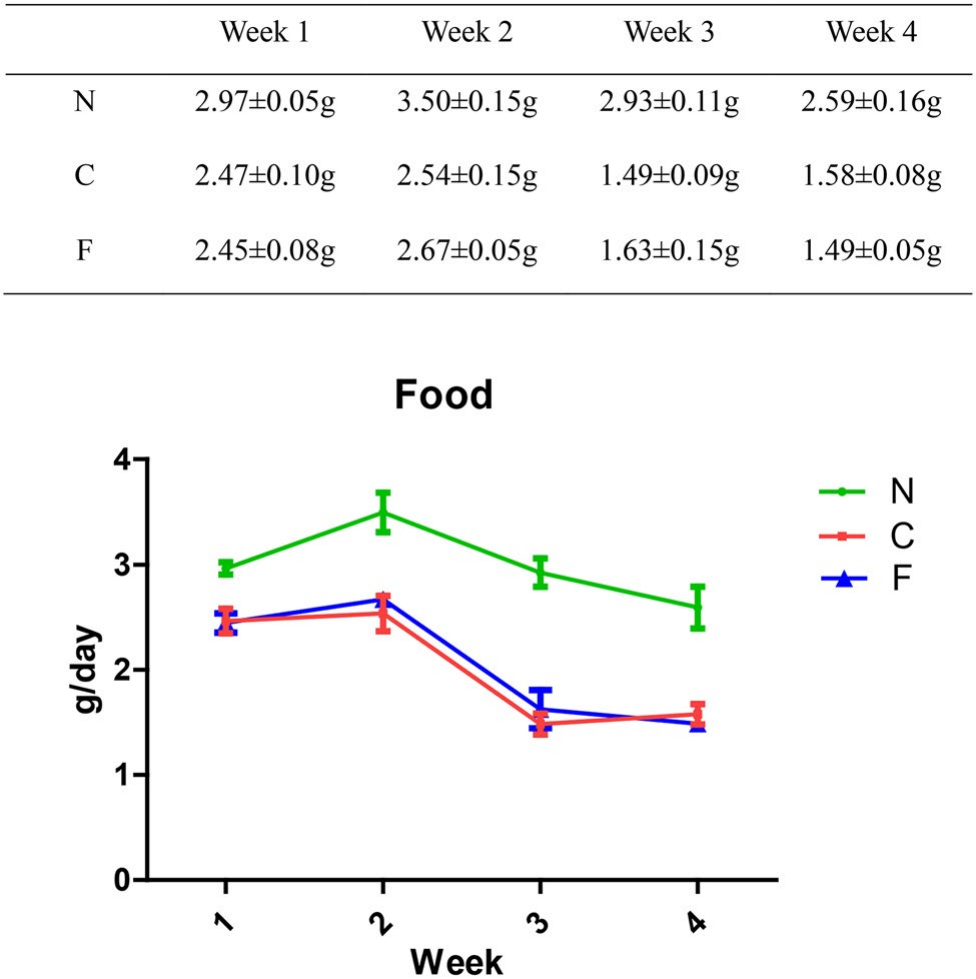
Effect of adenine and fecal bacteria transplantation on food intake in mice.

**Figure 4.**
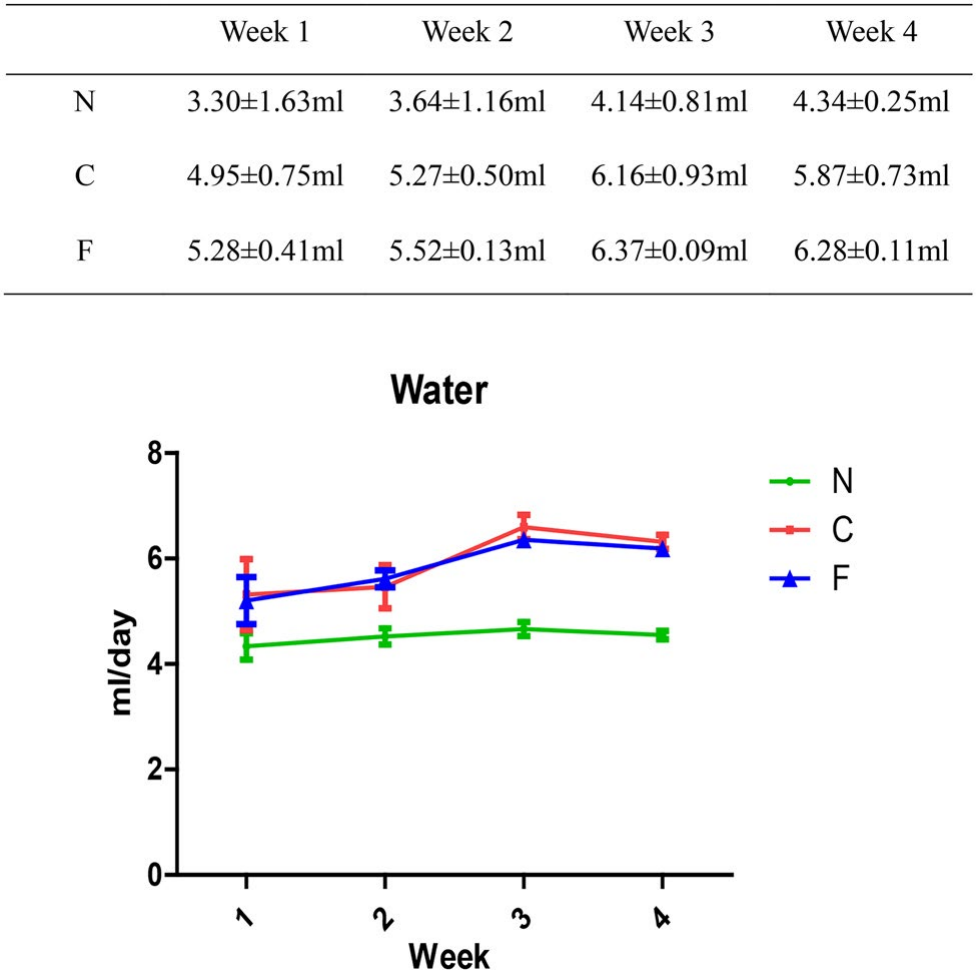
Effect of adenine and fecal bacteria transplantation on water intake in mice.

**Figure 5.**
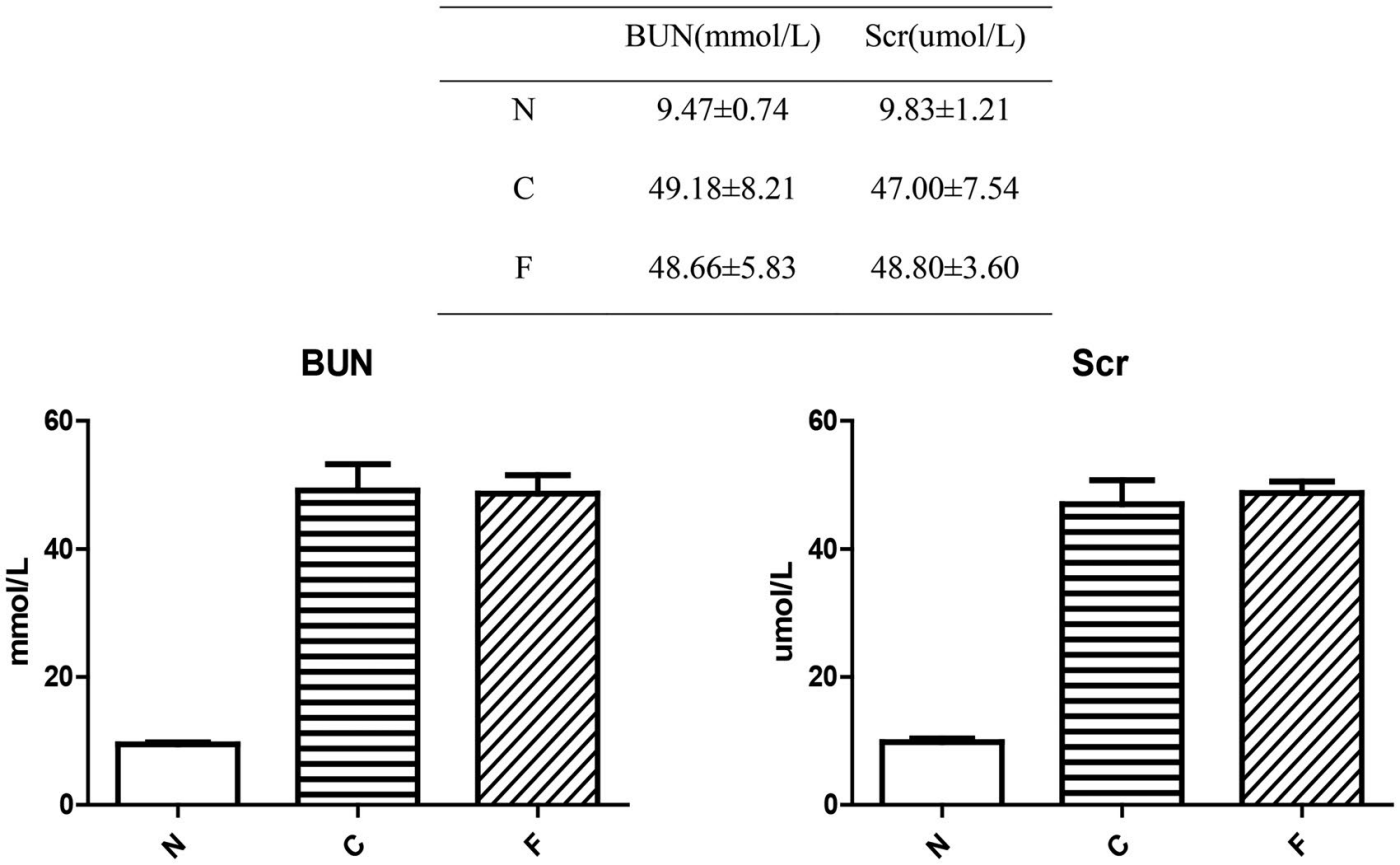
BUN level and creatinine level in mice after 4 weeks of modeling.

**Figure 6.**
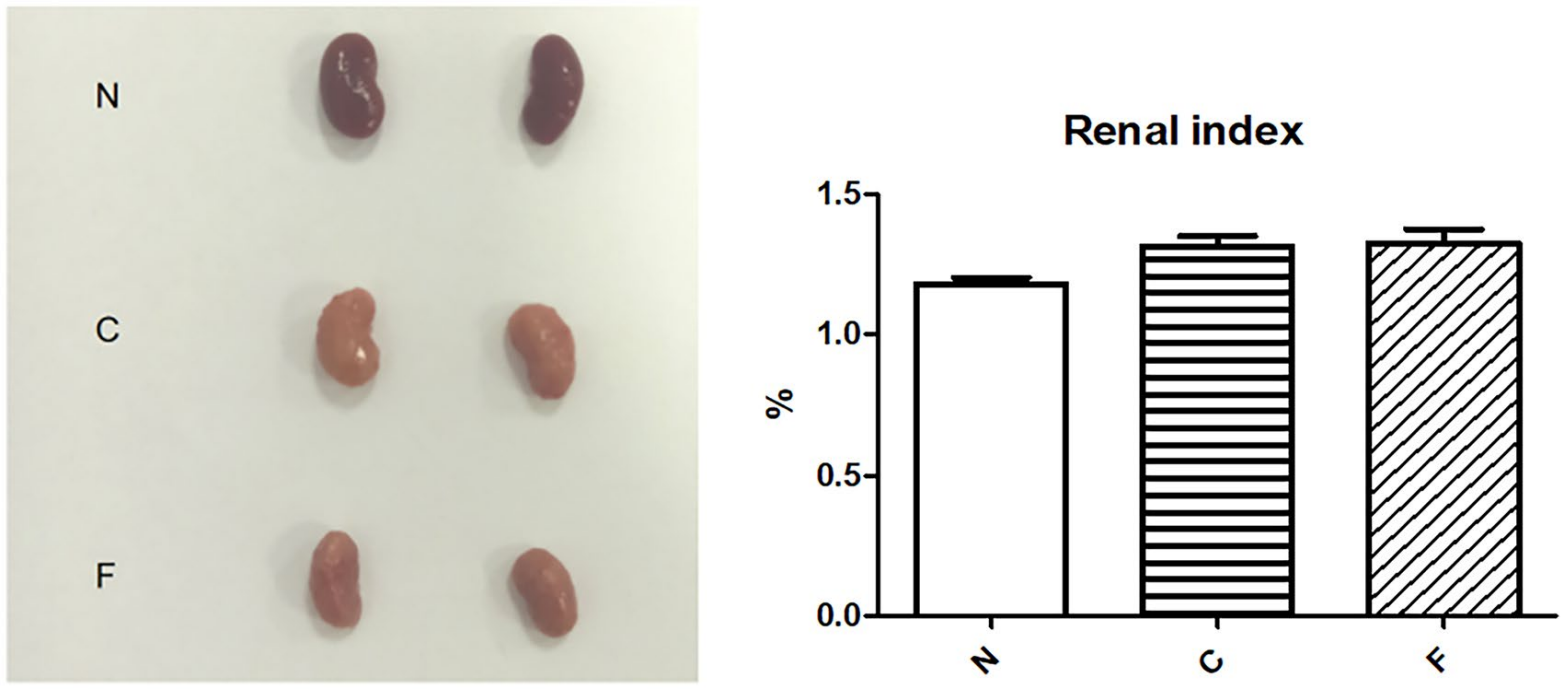
Anatomic appearance and renal index of mouse kidneys after 4 weeks of modeling.

**Figure 7.**
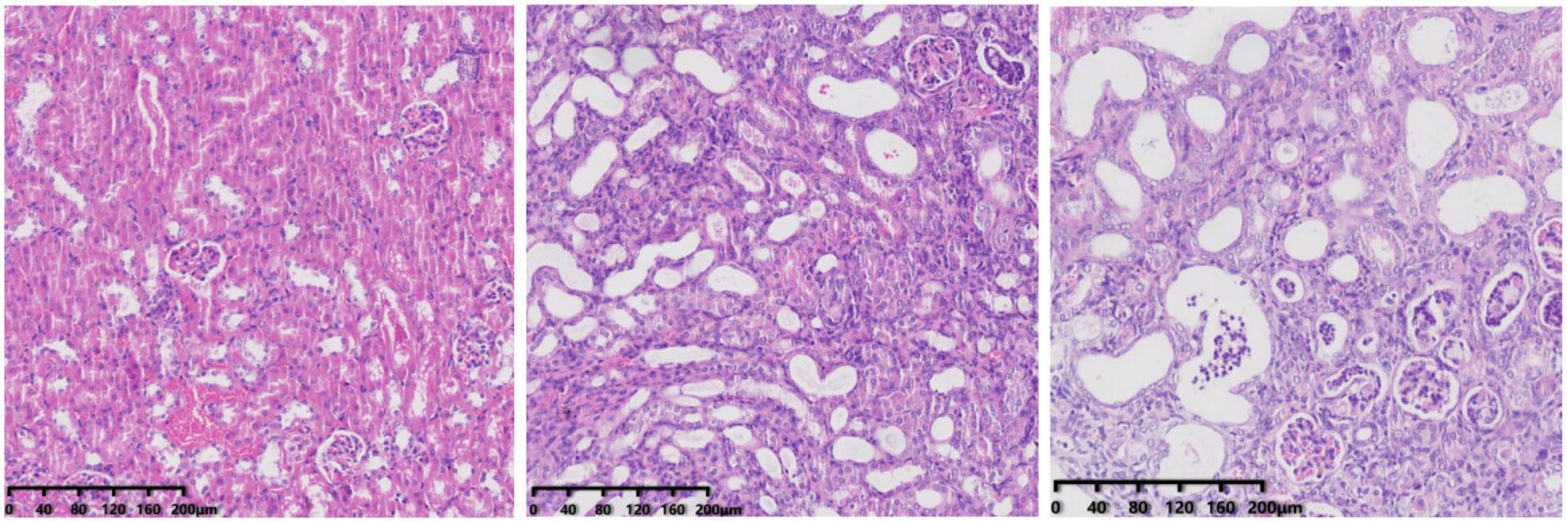
Renal pathology of mice after 4 weeks of modeling (from left to right, N, C, F).

We created a CRF model in mice induced by feeding chow containing 0.2% adenine for 4 weeks. Compared with control-group mice fed with normal chow, model-group mice showed normal bodyweight, normal diet, good mental health, good activity, shiny fur, sensitive responses, and normal defecation. Model-group mice gradually showed signs of emaciation (Figure 2), eating less (Figure 3), drinking more water (Figure 4), urinating more, feeling lethargic, decreased activity, matted fur, fur shedding, as well as squinting eyes, wet and cold tails, and dry and gray stools. After 4 weeks of modeling, biochemical indicators were tested: model-group mice showed a significant increase in BUN level and serum level of creatinine (Figure 5). The kidneys of model-group mice were atrophic, had a granular surface, and had an increased renal index (Figure 6). H&E staining showed that the glomeruli of normal-group mice were evenly distributed, with no atrophy or proliferation changes, no dilation of renal tubules, and no infiltration of inflammatory cells in the renal interstitium. Model-group mice showed significant dilation of renal tubules, infiltration of renal interstitial inflammatory cells, degeneration of renal tubular epithelial cells, necrosis of some renal tubular epithelial cells, and the appearance of a purulent cell type, with mild proliferation of renal cortical interstitial fibrous tissue (Figure 7). A mouse model of CRF was established. The success rate in establishing a CKD mouse model after four weeks of adenine feeding is 100%. This modeling method did not require surgery, was easy to operate, and did not cause death.

**Figure 8.**
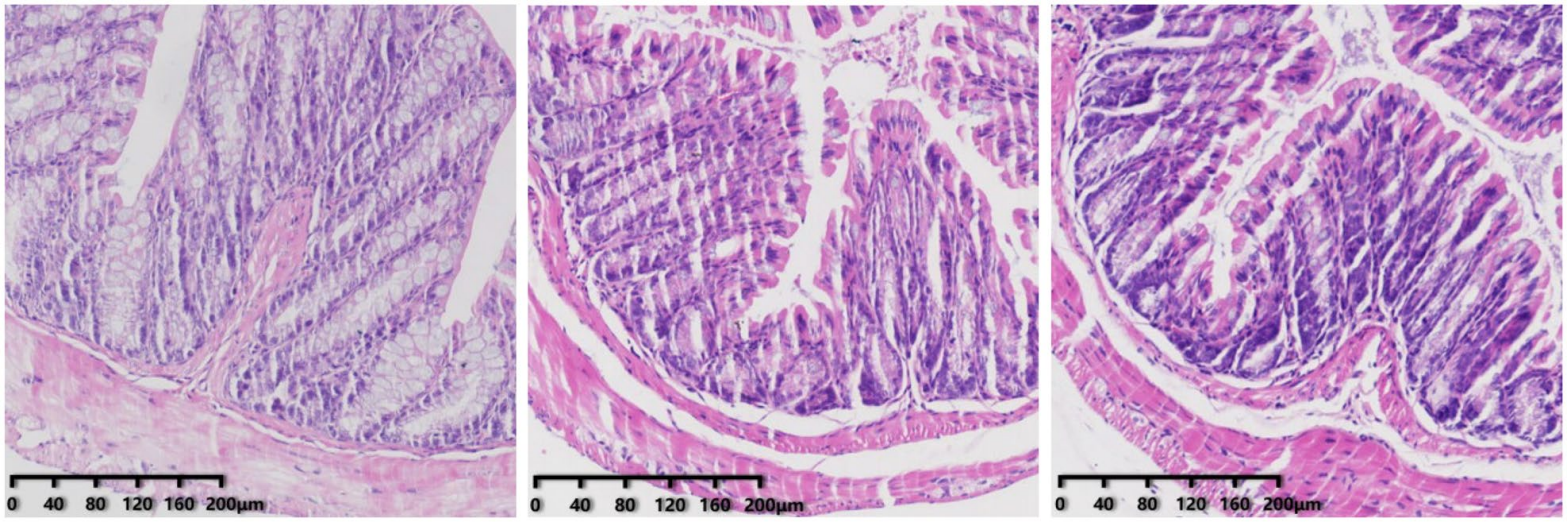
Effect of cefoperazone/sulbactam on mouse survival.

After establishment of the model, we injected (i.p.) different concentrations of cefoperazone/sulbactam into normal control mice and CRF mice. All CRF mice experienced grade-V seizures and eventually died, whereas normal control mice did not (Figure 8). These data indicated that cefoperazone/sulbactam could induce epilepsy and death in CRF mice, but had no effect on normal mice. Simultaneously, we observed that after intraperitoneal injection of cefoperazone/sulbactam, the feces of mice showed morphologic changes, with golden and moist colors. Therefore, we speculate that the GM may be involved in the epilepsy induced by cefoperazone/sulbactam in CRF mice.

Ritz proposed the concept of ‘enterorenal syndrome’ at the 2011 International Dialysis Conference. He stated that the occurrence of heart complications and other complications and a reduced chance of survival in patients undergoing hemodialysis were related to the inflammatory response caused by intestinal bacteria and endotoxins passing through the damaged intestinal mucosal barrier. Meijers et al. proposed the concept of the ‘intestine–kidney axis’. They revealed the relationship between CKD and the GM, whereby the latter and its metabolites have important roles [10].

The theory of the gut–kidney axis [11] suggests that patients suffering from CKD may experience GM disturbances during disease progression. This phenomenon would result in significant changes in the GM structure, including a decrease in the probiotics level and an increase in conditional pathogenic bacteria that can produce urinary toxins. This action exacerbates the accumulation of intestinal-derived urinary toxins in the blood, which cannot be cleared by the damaged kidneys in a timely manner. This scenario leads to further decline in renal function, ultimately forming a ‘vicious cycle’ between the intestine and kidneys. On the other hand, dysregulated GM can also disrupt the integrity of the colonic epithelial barrier. This phenomenon causes intestinal-derived urinary toxins and conditional pathogens to migrate into the bloodstream, activating the intestinal mucosal immune system, inducing systemic micro-inflammatory reactions, and exacerbating kidney damage. These pathologic changes can induce further accumulation of urinary toxins, leading to systemic inflammation, immune disorders, metabolic disorders, and cardiovascular events in patients with CKD. The intervention methods for treating chronic kidney failure include: (i) a low-protein diet that reduces toxin production by limiting the intake of the precursors of urinary toxins; (ii) microbial preparations to restore the GM balance; (iii) adsorbent therapy to strengthen the treatment of toxins in the body.

Increasing numbers of studies have found interactions between the GM and organs. Vallianou et al. pointed out that GM plays an important part in CKD and hypertension, and proposed the concept of the ‘brain–gut–kidney axis’ [12] (i.e., complex interactions between the gut, microbiota, kidney, and brain).

How the GM causes epilepsy is not clear. The GM regulates the gut and CNS through neurotransmitters and multiple pathways (e.g., endocrine, immune, and metabolic). Research has shown that the higher the diversity of the GM, the more stable is the immune function, and the lower the diversity of the GM, the more unstable is its immune function. Hence, the GM can alter the immune function of the body.

**Figure 9.**
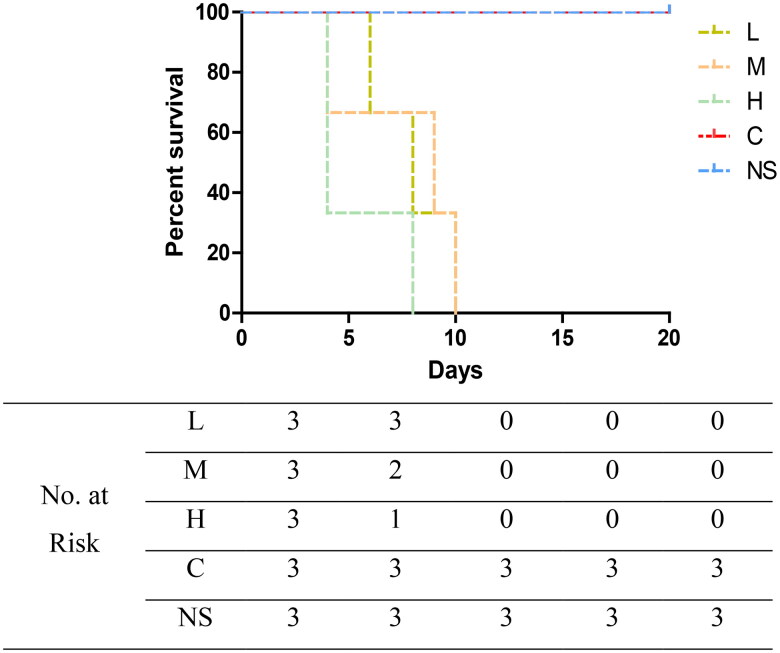
Effect of fecal bacteria transplantation on the survival of mice suffering from epilepsy.

We extracted and prepared a suspension of fecal microbiota, and then administered it to CRF mice to prepare an FMT model. FMT had no significant effect on the bodyweight, food intake, or water intake of CRF mice, and did not change the state of polyuria or mental depression in mice. However, compared with the dry and dark feces of CRF mice, the feces of mice after FMT intervention became moist and yellow. CRF mice and FMT-intervened CRF mice were injected (i.p.) with cefoperazone/sulbactam, and both groups developed epilepsy. However, after FMT intervention, the time of epilepsy onset and death in mice was delayed. Early FMT intervention resulted in more mice surviving (Figure 9). We suggest that FMT can delay the onset of epilepsy in CRF mice induced by cefoperazone/sulbactam, and the earlier the intervention, the better is the effect.

**Figure 10.**
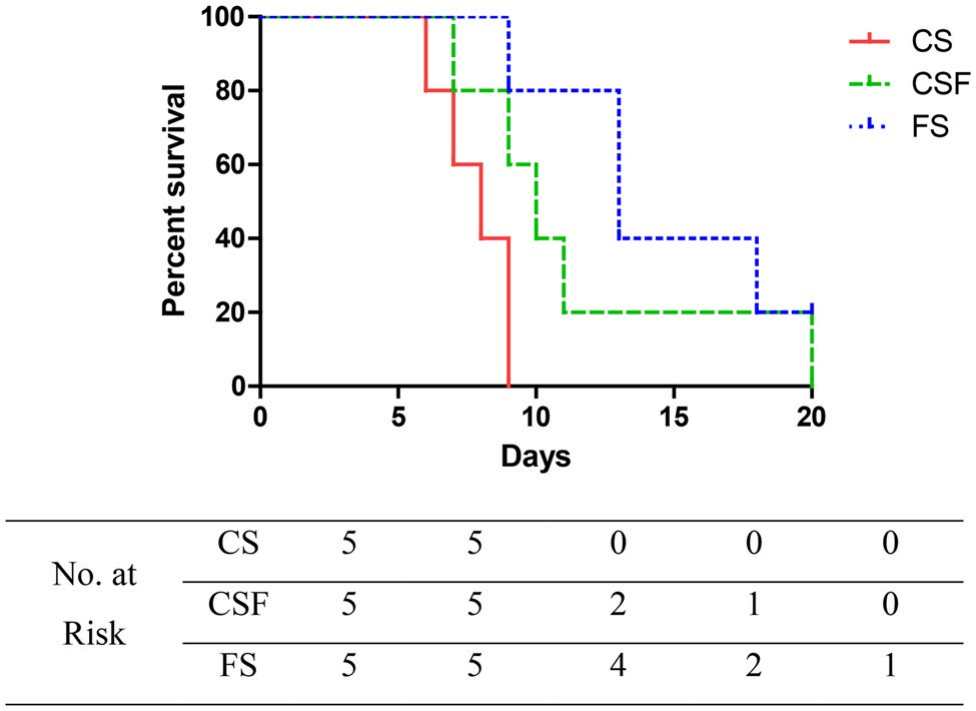
Colon pathology in mice after 4 weeks of modeling (from left to right, N, C, F).

**Figure 11.**
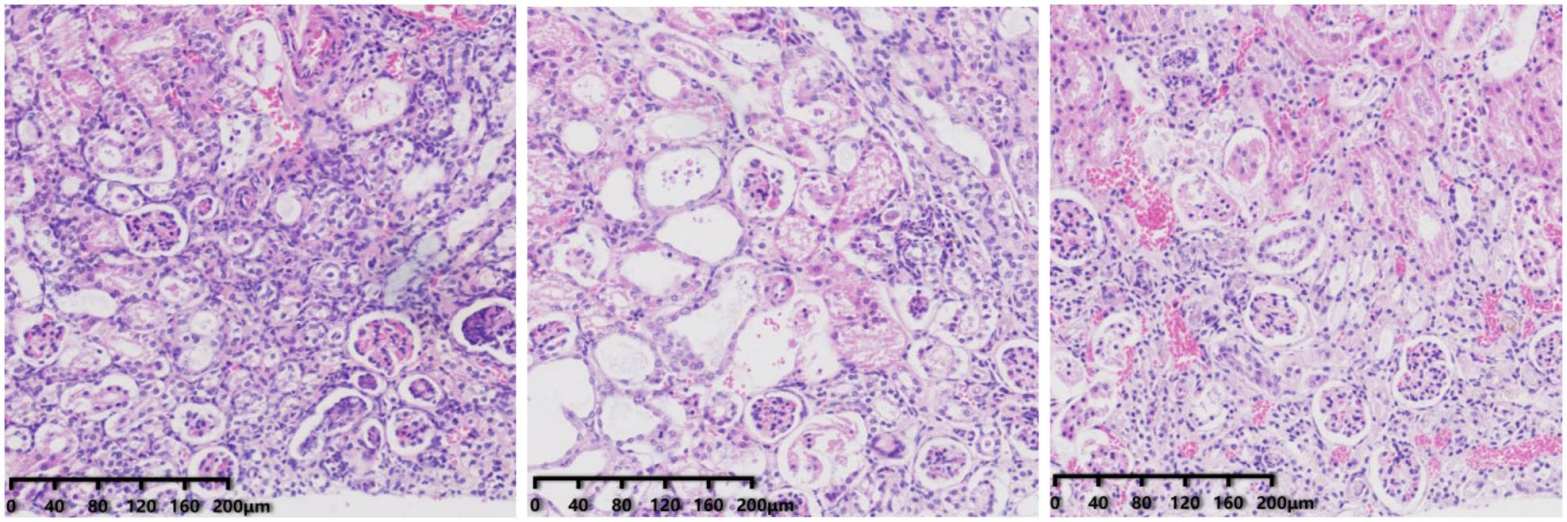
Colon pathology after FMT intervention (from left to right, CS, CSF, FS).

We obtained the colon of mice for H&E staining. There was no significant difference between the colon of CRF mice and FMT mice and normal mice (Figure 10). However, after injection of cefoperazone/sulbactam, there were varying degrees of villus detachment as well as separation of the mucosal layer and submucosal layer (Figure 11). These results indicated that cefoperazone/sulbactam could affect the structure of the mouse colon. Early FMT intervention could reduce this damage.

**Figure 12.**
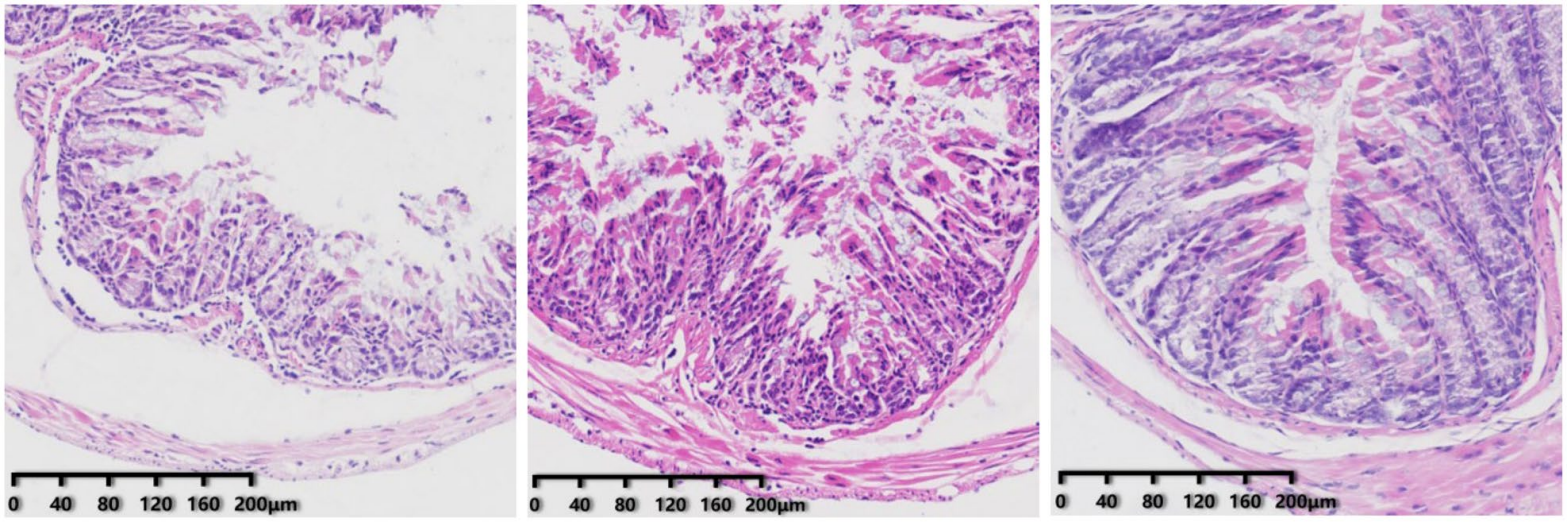
Renal pathology after FMT intervention (from left to right, CS, CSF, FS).

In addition, we obtained the kidneys of mice for H&E staining. After injection of cefoperazone/sulbactam, the kidneys of mice showed infiltration of inflammatory cells, while mice intervened with FMT had fewer inflammatory cells. These results indicate that cefoperazone/sulbactam may induce renal inflammation in mice, and FMT intervention can reduce the occurrence of inflammation (Figure 12).

**Figure 13.**
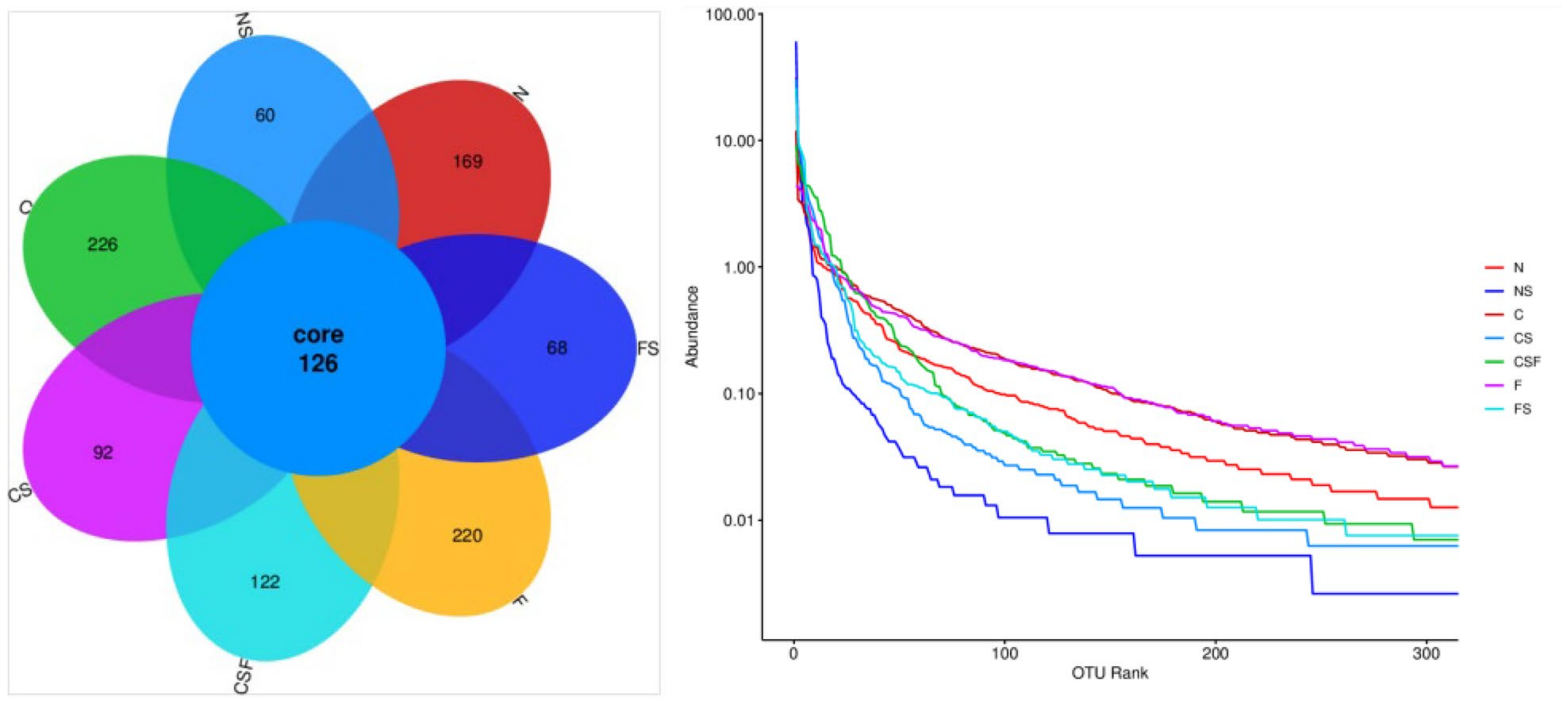
Microbial diversity of samples.

**Figure 14.**
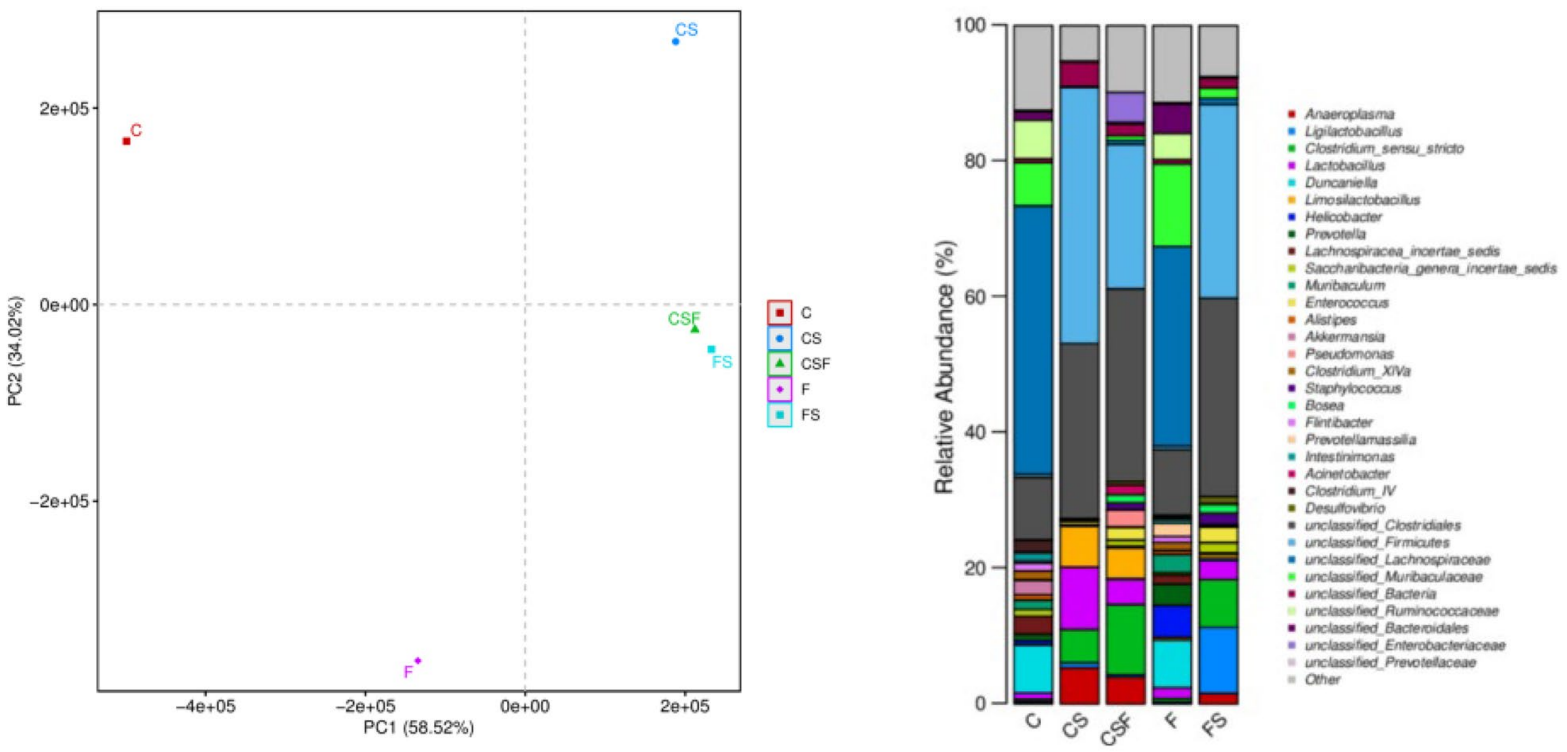
Bacterial distribution of each group of samples.

We collected the feces of mice in each group and conducted 16S RNA sequencing. CRF mice injected with cefoperazone/sulbactam had reduced microbial diversity (Figure 13), disrupted GM, an increased proportion of *Anaeroplasma*, *Clostridium*, *Lactobacillus*, and *Limosilactobacillus*, but a reduced proportion of bacteria of the genera *Duncaniella*, *Enterococcus*, *Staphylococcus*, and *Bosea*. After FMT, the diversity of bacteria in mice increased, and the abundance of *Anaeroplasma*, *Clostridium*, *Lactobacillus*, and *Limosilactobacillus* decreased, whereas the proportion of bacteria of the genera *Duncaniella*, *Enterococcus*, *Staphylococcus*, and *Bosea* increased (Figure 14). These data also suggested that abnormal immune function induced by a GM disorder could be one of the reasons for epilepsy in patients with renal insufficiency, and the bacterial populations stated above may be involved.

Our study had six main limitations. First, the kidney and colon of mice were stained only with H&E, and further analyses of the proportion and distribution of inflammatory cells were not possible. Second, the mechanism of action of FMT is not yet clear, and there is no universal super stool. The efficacy of FMT varies among different pathological conditions. Third, due to the small amount of mouse feces, there was only one sample per group for 16S RNA sequencing of fecal bacteria, which could not be analyzed statistically. The suspension of fecal bacteria was not purified, so we could not identify which bacteria were involved in the occurrence and development of epilepsy in mice. Fourth, we conducted only animal experiments and did not collect human feces for 16S RNA sequencing. Fifth, cefoperazone/sulbactam has the potential to cause coagulopathy, and mouse deaths may be related to coagulopathy. In this experiment, we did not test the coagulation function of the mice. We will complete it in further experiments to exclude the impact of coagulation function on mouse death. Finally, in the later stage of the experiment, it is planned to further clarify whether the epilepsy in mice was induced by cefoperazone or sulbactam.

## Data Availability

The datasets generated and analyzed during the current study are available from the corresponding author on reasonable request.
